# Long-Term Macular Vascular Changes after Primary Rhegmatogenous Retinal Detachment Surgery Resolved with Different Tamponade or Different Surgical Techniques

**DOI:** 10.3390/life12101525

**Published:** 2022-09-30

**Authors:** Matteo Gironi, Rossella D’Aloisio, Tommaso Verdina, Chiara Vivarelli, Riccardo Leonelli, Shaniko Kaleci, Lisa Toto, Rodolfo Mastropasqua

**Affiliations:** 1 Ophthalmology Clinic, University of Modena and Reggio Emilia, Azienda Ospedaliero-Universitaria di Modena, 41122 Modena, Italy; 2 Ophthalmology Clinic, Department of Medicine and Science of Ageing, University G. D’Annunzio Chieti-Pescara, 66100 Chieti, Italy; 3 University of Modena and Reggio Emilia, Azienda Ospedaliero-Universitaria di Modena, 41122 Modena, Italy

**Keywords:** rhegmatogenous retinal detachment, optical coherence tomography angiography, vitrectomy, silicon oil, macular vessel density

## Abstract

**Simple Summary:**

Primary rhegmatogenous retinal detachment is an acute threat to visual impairment due to a retinal break that allows the passage of vitreous fluid into the subretinal space. Although it is clear that functional results are influenced by retinal detachment extension and surgical timing, we do not have definitive indications on post-operative changes in macular microcirculation and how they might affect visual performance. The study aims to evaluate the long-term macular vascular changes and their correlation with functional recovery in patients successfully treated for rhegmatogenous retinal detachment. We found a reduction of the vascular density in the operated eyes, not influenced by surgical techniques, independently from the pre-operative detachment extension. However, we found that functional recovery is influenced by different intraoperative choices. In conclusion, such visual acuity and microvascular changes can be considered biomarkers that highlight the relevance of careful management of this sight-threatening disease.

**Abstract:**

Background: The aim of this study was to assess long-term macular vascular changes and their correlation with functional recovery in patients successfully treated for Macula-ON and Macula-OFF rhegmatogenous retinal detachment (RRD). Methods: This retrospective observational study included 82 eyes of 82 patients who received primary successful retinal detachment surgery, 33 Macula-ON and 49 Macula-OFF. Superficial and deep capillary plexuses (SCP and DCP) were evaluated by optical coherence tomography angiography (OCTA), and were correlated with visual acuity (VA), surgical technique and tamponade at 12 months after surgery. The fellow eyes were used as controls. Results: At 12-month follow-up, there was a significant decrease in the vessel density (VD) in the SCP in the operated eyes compared to control eyes (*p* < 0.05) in both the Macula-ON and Macula-OFF groups. Vessel length density (VLD) decrease in SCP was more extended in the Macula-OFF group. No difference in the DCP perfusion parameters was found, compared to controls. Subgroup analysis dependent on the type of surgery or tamponade showed no significant differences of VD and VLD. An inverse correlation was found between the SCP VD and the duration of silicone oil (SO) tamponade (*p* = 0.039). A significant correlation was observed between parafoveal SCP VD and final best corrected visual acuity (BCVA) (*p* = 0.028). The multivariate linear regression analysis showed that only the type of tamponade was significantly correlated with the final BCVA in the Macula-ON group (*p* = 0.004). Conclusions: Our study described long-term perfusion changes in RRD after surgery, with lower SCP VD and VLD in the operated eyes compared to the fellow ones, not influenced by type of surgery or tamponade. The choice of tamponade and SO removal timing may affect functional outcomes, especially in Macula-ON RRD. In conclusion, such functional and perfusion changes can be considered biomarkers that highlight the relevance of careful management of this sight-threatening disease.

## 1. Introduction

Rhegmatogenous retinal detachment (RRD) is defined as the separation of the neurosensory retina from the retinal pigment epithelium (RPE), triggered by retinal breaks which allows vitreous fluid to escape into the subretinal space. RRD is an acute threat to visual impairment which requires early surgical management. The surgical techniques can be different, including scleral buckling (SB) and pars plana vitrectomy (PPV) with internal tamponade such as gas or silicone oil (SO). Despite the high anatomical restoration after vitreoretinal surgery, some patients continue to have poor visual recovery [1]. The main preoperative predictive factor for visual recovery is the macular involvement. Optical coherence tomography has been widely used to study the influence of type of surgery, tamponade and extension of retinal detachment (RD) on functional and anatomical retinal recovery [2,3].

Optical coherence tomography angiography (OCTA) is a non-invasive method to study the macular microcirculation, with good reproducibility and repeatability [4,5,6]. It produces clear, depth-resolved visualization of the retinal and the choroidal microvasculature without intravenous fluorescent dye. OCTA can provide binarized and skeletonized images for microvasculature evaluation in the superficial capillary plexus (SCP), in the deep capillary plexus (DCP) and in the choriocapillaris plexus (CCP). Recently, macular microvascular parameters, revealed on OCTA scan, were proposed as a new biomarker associated with functional and anatomic recovery after RRD repair. An impairment on retinal microvasculature after vitreoretinal surgery, in terms of enlargement of the foveal avascular zone (FAZ) area and decrease in retinal vessel density, emerged by literature [7]. This is in accordance with previous fluorescein angiography-based studies, which described a reduced and slow retinal blood flow in patients with RD because of a growth in peripheral resistances [8,9].

The aim of this study was to evaluate foveal microvascular structure and investigate the significance of long-term vascular changes in the eyes with successfully reattached RRD with different surgical techniques (SB or PPV and gas or SO tamponade). We have also compared perfusion/architectural parameters with functional findings.

## 2. Materials and Methods

### 2.1. Design and Setting of the Study

This retrospective observational study was conducted at the Ophthalmology Clinic of University of Modena and Reggio Emilia (Italy). This work was approved by the Ethics Committee of the Vasta Emilia Nord Area (protocol AOU n. 0016643/21, study number 256/2021/OSS/AOUMO SIRER ID 2232) and was conducted following the principles of the Declaration of Helsinki. 

### 2.2. Participants

The study sample consisted of patients who underwent RRD surgery in the period between June 2018 and March 2021. Inclusion criteria were primary unilateral RRD, presence of successful retinal reattachment after single surgical intervention (SB or PPV with gas or SO tamponade), and absence of macular pathologies that could potentially impair visual function. We defined patients with an RRD not involving the fovea as the Macula-ON group and those with an RRD with foveal detachment as the Macula-OFF group. Successful retinal reattachment was considered if all the breaks were supported and adequately sealed with retinal laser and the macula was adequately attached. 

Patients with severe proliferative vitreoretinopathy (PVR) were excluded. Other exclusion criteria were previous surgical interventions (other than uneventful cataract surgery), re-detachment, persistent subretinal fluid and prior retinal detachment in the fellow eye. 

Patients who met the inclusion criteria underwent a complete ophthalmic examination for both eyes, including BCVA, intraocular pressure, slit-lamp biomicroscopy, fundoscopy, spectral-domain (SD)-OCT and OCTA, along with a complete medical history. For all patients, the fellow eyes were considered as the control group. Pre-operative and intra-operative data collected from the medical records were age, sex, hypertension, diabetes, BCVA, retinal detachment extension and surgical procedure information (such as combined phaco-vitrectomy) and intra-operative retinal photocoagulation laser application.

### 2.3. Surgical Procedure 

25G 3-port pars plana vitrectomy was performed in 70 eyes (PPV) by the same expert surgeon (RM). After core vitrectomy, a complete vitreous base removal was performed. The surgeon removed as much of the peripheral vitreous as possible, thus releasing vitreous traction on the retinal break. Subretinal fluid was aspirated using a flute needle during the fluid/air exchange. Endolaser photocoagulation was applied around the break(s) or 360° retinal photocoagulation was performed in the presence of multiple breaks in many quadrants. After that, gas (SF6 or C3F8) or SO tamponade was injected in vitreous chambers.

Scleral buckle (SB) was performed in 12 eyes (SB group) by the same surgeon (RM). Segmental radial or circumferential buckles were sutured above the retinal breaks and external subretinal fluid drainage was performed. Finally, an intravitreal expansible gas injection was performed.

Young phakic patients, without posterior vitreous detachment, preferably underwent episcleral surgery, otherwise PPV was the first choice for surgical management. Combined phacoemulsification, aspiration, and intraocular lens implantation were performed at the discretion of the surgeon in cases of phakic patients

At the time of data collection, all eyes underwent silicone oil removal surgery, or complete resorption of the gas bubble was ascertained.

### 2.4. Functional Parameter: Best-Corrected Visual Acuity

The best-corrected visual acuity (BCVA) was assessed using Snellen’s visual acuity chart. The recording of BCVA was performed preoperatively and during the postoperative visit. The BCVA was converted from Snellen’s scale to the logarithm of the minimum angle of resolution (logMAR) units for the purpose of statistical analysis. 

### 2.5. Perfusion Parameters: OCTA Image Acquisition and Analysis

**Figure 1:**
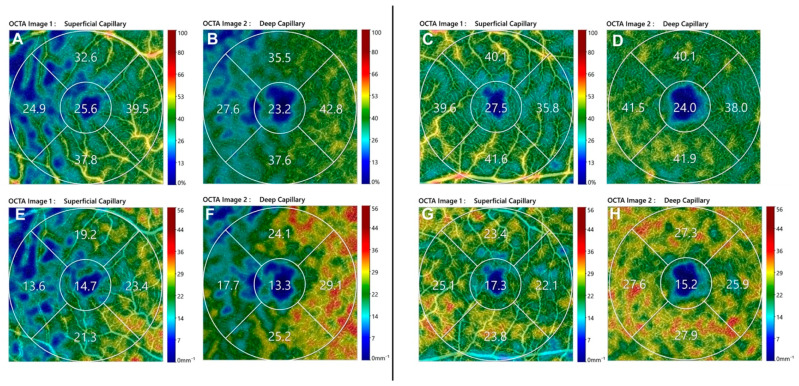
Optical coherence tomography angiography 3 × 3 images of post-operative perfusion parameters in Macula-ON (left) and Macula-OFF (right) rhegmatogenous retinal detachment eye. (Above) SCP VD of Macula-ON (**A**) and Macula-OFF (**C**) and DCP VLD of Macula-ON (**B**) and Macula-OFF (**D**); (Below) DCP VD of Macula-ON (**E**) and Macula-OFF (**G**) and DCP VLD of Macula-ON (**F**) and Macula-OFF (**H**). SCP, Superficial Capillary Plexus; DCP, Deep Capillary Plexus; VD, Vessel Density; VLD, Vessel Length density.

3 × 3 mm OCTA scans of SCP and DCP were performed using Canon OCT HS100 angiography^®^ trough-dilated pupils. The OCTA images of the SCP and DCP were binarized and then, skeletonized by integrated-images-processing system software. Vessel density (VD) calculated on binarized image was considered as the percentage of white pixels in the region by percent (%). On skeletonized images, the lines of a binary image were transformed into thin lines (1 pixel of thickness) and the value obtained by dividing the sum of the length of the thin lines in the region by the area by “mm-1” to represent the vessel length density (VLD). All the above-mentioned image processing was performed by the software (OCT-HS100 Angio Expert AX^®^). Predefined automatic segmentation for the retinal vascular plexuses was performed by the integrated software (RX Capture for OCT-HS100^®^). (Figure 1) An all-images review was performed by two vitreoretinal specialists (R.DA. and M.G.). At the study examination, VD and VLD were automatically calculated in a fovea-centered circle area (1 mm diameter around the foveola) and parafoveal area (annular area extending between 1 and 2.5 mm diameter, centered on the foveola) was segmented in four quadrants (superior, nasal, inferior and temporal), then density parameters were recorded for each of them. Furthermore, the mean values of VD and VLD in the parafoveal area and the whole macula area in SCP and DCP were recorded for each patient. In addition to the OCTA images, OCT images were also acquired for all the patients enrolled in the study. The central macular thickness (CMT) was measured with retinal thickness map of SD-OCT, in Macula 3D mode.

### 2.6. Statistical Analysis

Continuous variables were presented as mean values ± standard deviation (SD). The categorical data were described as the frequency counts and percentages. The quantitative data fitted a normal distribution, and a paired t-test (Student’s *t* test) was used to compare ocular parameters of RRD eyes with the fellow eyes. Analysis of variance (ANOVA) was performed. Bivariate correlations and possible associations between vascular parameters and post-operative BCVA were analyzed by applying Pearson correlations. To identify the factors related to BCVA in the studied eyes, the potential determinants were tested using univariate and multivariate linear regression analyses. All the *p* values were considered statistically significant when the values were <0.05. Statistical analyses were performed with SPSS (IBM Corp. Released in 2017. IBM SPSS Statistics for Windows, Version 25.0. Armonk, NY, USA: IBM Corp) and STATA14 (Stata. Release 14. Statistical Software. College Station, TX, USA: StataCorp LP).

## 3. Results

A total of 82 patients (50 men and 32 women) with mean age of 63 ± 12 years were included in the study. Demographics, ocular data and characteristics of study group are reported in Table 1. 

SB was performed in 12 eyes and PPV in 70 eyes. All the patients in the PPV group patients were pseudophakic at the time of study recruitment. The mean follow-up period was 13.1 ± 2 months. In the PPV group the mean duration of SO tamponade was 4.81 ± 1.8 months. Eight eyes (9.8%) experienced increased IOP during the post-operative period; thus, requiring topical anti-glaucoma eyedrops. No eyes required glaucoma surgery. 

The Macula-ON group’s pre-operative and post-operative visual acuity were significantly better than in the Macula-OFF group. A post-operative significant improvement in terms of BCVA was observed in the Macula-OFF RRD group (Pre-Op BCVA 1.248 ± 0.86 vs. Post-Op BCVA 0.210 ± 0.33; *p* = 0.00). The Macula-ON group had a non-significant improvement of BCVA at follow-up (*p* = 0.245).

A surgical technique-based subgroup analysis showed that final BCVA was not influenced by type of surgery. The sub-analysis dependent on macula involvement and tamponade has shown that the Macula-ON group’s eyes filled with gas had a better final BCVA (0.058 ± 0.10 logMAR) than those with SO (0.333 ± 0.41 logMAR; *p* = 0.003). The multivariate linear regression analysis showed that only the type of tamponade (*p* = 0.004) was significantly correlated with the final BCVA in the Macula-ON group, as showed in Table 2. Patients who developed intraretinal cysts had the worst final BCVA in comparison to patients who did not develop intraretinal cysts (0.131 ± 0.28). 

Compared to healthy fellow eyes, RRD eyes had a significant lower SCP VD and VLD (*p* < 0.05) for all quadrants, but no statistically significant difference in DCP.

**Figure 2:**
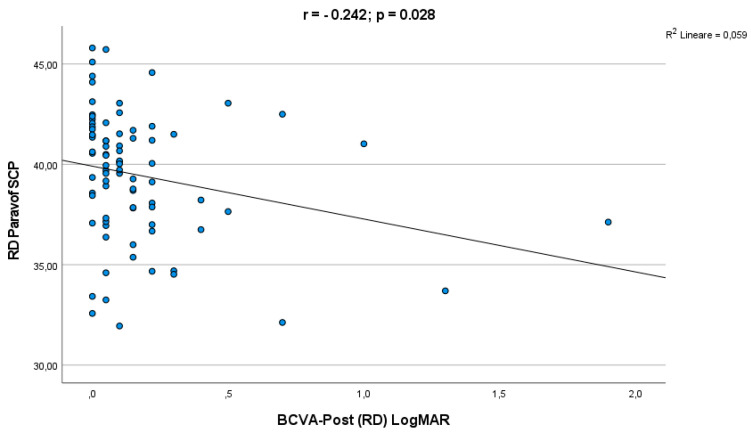
Correlation analysis between final BCVA and parafoveal SCP VD.

A significant correlation was observed between final LogMAR BCVA and parafoveal SCP VD (r = −0.242; *p* = 0.028) (Figure 2).

### 3.1. Macula-ON Group

Compared to healthy fellow eyes, RRD eyes had a lower SCP VD in the central, nasal, and superior quadrant and a lower SCP VLD only in the central quadrant. There was no statistically significant difference in VD between treated eyes and controls in all DCP segments (Table 3).

### 3.2. Macula-OFF Group

Compared to healthy fellow eyes, RRD eyes had a lower SCP VD and VLD in all the segments. There was no statistically significant difference in VD and VLD between treated eyes and controls in the DCP. (Table 3) The Macula-OFF group had a lower foveal, parafoveal and total VD in SCP and DCP in comparison to the Macula-ON group, although it was not statistically significant. 

### 3.3. Surgery Subgroup Analysis

**Figure 3:**
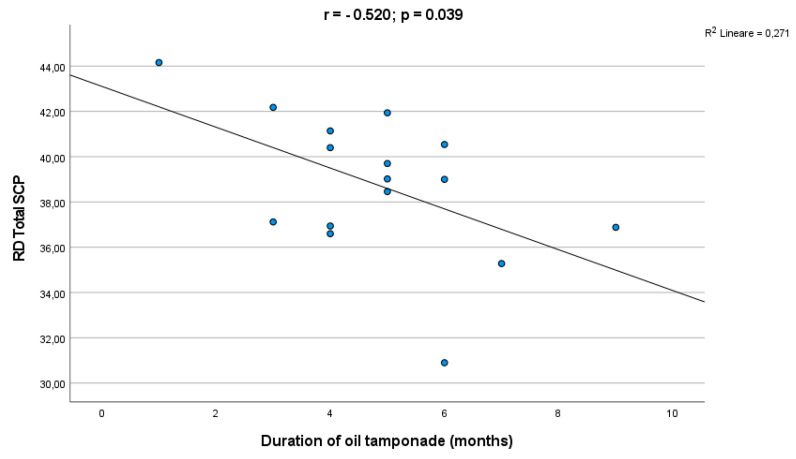
Correlation analysis between the silicone oil tamponade duration and the SCP VD.

The subgroup analysis between PPV and SB showed lower VD and VLD in the SCP in comparison to fellow eyes for both groups. Either eyes filled with gas or SO had a lower VD and VLD in the SCP in comparisons to fellow eyes. The sub-analysis dependent on the type of surgery or tamponade has shown that there was no significant difference in the foveal, parafoveal and whole VD of the SCP and DCP either between PPV and SB or gas and SO groups. In the correlation analysis an inverse correlation was found between the SCP VD and the duration of SO tamponade (r = −0.520, *p* = 0.039) (Figure 3).

### 3.4. OCT Findings

No differences were found between the two groups (Macula ON and OFF) in terms of CMT (*p* > 0.05).

Post-operatively, eyes filled with SO had a greater CMT (349.53 ± 76.58 µm) in comparison to eyes filled with gas (307.97 ± 32.56 µm, *p* = 0.002). Patients who developed intraretinal cysts had a significantly greater CMT (367.58 ± 81.70 µm) in comparison to patients who did not develop cysts (308.00 ± 33.40 µm, *p* = 0.000). In the Macula-OFF group eyes filled with SO the CMT was statistically greater than in those filled with gas. In the SO group 47% (8/17) of subjects developed CME, although CME was revealed only at 6% (4/65) in the gas group.

## 4. Discussion

In the present study we aimed at assessing macular perfusion changes that occurred after RRD, investigating the perfusion parameters of Macula-OFF and Macula-ON RRD eyes treated with different surgical techniques (PPV or SB) and different tamponade (gas or silicone oil).

As expected in our cohort, a statistically significant post-operative improvement of BCVA was found in the Macula-OFF group [10,11] given that macular involvement was considered as the main factor correlated with final visual prognosis [12].

In detail, the multivariate regression analyses showed that only the type of tamponade was significantly correlated with the final BCVA in the Macula-ON group, as eyes filled with gas had a better final BCVA then those filled with SO and this is in line with some previous studies [13,14]. 

Moreover, visual acuity was negatively related to parafoveal SCP VD, suggesting that as the perfusion parameter decreased after surgery, a concomitant lowering of BCVA was detected, similarly to the RVO condition [15]. These events may indicate that retinal hypoperfusion due to an acute decreased and slowing of retinal blood flow in patients with retinal detachment induced microarchitectural modifications for several months after the surgery with the consequent permanent functional impairment of retinal ganglion cells and photoreceptors, leading to long-term deterioration of the visual acuity [16,17,18,19,20].

Our study emphasizes the concept of a reduced retinal perfusion after RRD surgery, as we found a significant differences in SCP VD compared to control eyes at 12-month follow-up. 

The mechanism of retinal perfusion changes after RRD repair is still controversial. A fair number of studies with a follow-up no longer than 8 months were investigated. Reduction of VD in retinal capillary plexuses in comparison to fellow eyes at post-operative follow-up was usually reported in literature, with more significant evidence in Macula-OFF condition [11,21,22,23,24,25]. In contrast, Hong et al. found that the SCP and DCP VD in the operated eyes did not differ significantly from the fellow eyes for both the Macula-ON and Macula-OFF groups, at 6 months follow-up [26]. In some cases, a progressive increase in VD has been reported over the follow-up time [21,27]; on the contrary Jiang J et al., in their retrospective study, found a decreased VD at 16 wks post-operatively, after an initial increase during the first 12 wks. Unfortunately, our study could not evaluate this aspect as a single long-term follow-up was performed [28]. Long-time follow-up studies are more suitable to be compared to our findings. The first was conducted by Bonfiglio et al., who reported a lower parafoveal superficial VD and foveal and parafoveal deep VD in the Macula-OFF group compared to the contralateral eye at 12-month follow-up [29]. In the same study only parafoveal deep VD reduction was detected for Macula-ON. In accordance with our reports, Hassanpoor et al., in a small sample of Macula-OFF RRD (24 eyes), found that the parafoveal vessel density of SCP was lower in the operated eyes, with no significant changes in DCP in comparison to fellow eyes [30]. Another long-term follow-up study confirmed the reduction of VD associated to vitreoretinal surgery for RRD [31]. Resc M et al. found that SCP VD was predominantly affected after long-term (>12 months) post-operative time, reinforcing our findings [32].

We found also a reduced VLD in the SCP, in comparison to controls, more extensive in the Macula-OFF group than in the Macula-ON one. This discrepancy between the two groups may indicate that in the Macula-OFF group the smaller capillaries were more affected by RDD, in which the damage mainly manifests itself in a lower density of the VLD, probably due to a more extensive rarefaction of retinal vessels, if compared with the Macula-ON condition [33,34,35]. Indeed, the subretinal fluid could inhibit the provision of oxygen from the choriocapillaris to the outer retinal layers, triggering tissue hypoxia and release of cytokines and other inflammatory mediators [16,17,18]. These events may produce a persistent hypoperfusion for several months after the surgery and a permanent functional impairment of retinal ganglion cells and photoreceptors. VLD was previously correlated with macular ganglion cell/inner plexiform layer (mGCIPL) status [36]. An important parallel can be found in the study by Menke M et al. where GCL-IPL thickness in Macula-OFF successfully repaired RRD was significantly reduced in long follow-up patients (>8 weeks), but not in the short ones [37]. This supports the hypothesis that the functional damage of Macula-OFF patients had an anatomical equivalent in the internal retinal layers. 

In our cohort of patients, we did not find any differences in terms of VD with respect to the type of tamponade, similarly to Resc M. et al.’s findings [32].

We observed an inverse correlation between the SCP VD and the duration of SO tamponade; likewise previous studies showed that long-time SO tamponade could lead to a decrease in post-operative macular VD [28,38]. In addition, our study detected no differences of the VD according to the type of surgery. Otherwise, Nam et al. found that the PPV-operated eyes had a significantly lower parafoveal VD than the SB, postulating that there was more damage and a slower recovery in the PPV group [31].

Considering the absence of significant differences in OCTA-studied parameters in Macula-ON eyes between SO and gas groups, we could hypothesize a not primarily ischemic-pattern-retinal impairment for the explanation of the above-mentioned worst BCVA in SO filled eyes. Loss of retinal sensitivity might be led by toxic insult, photo-toxicity and failure of potassium siphoning by Muller cells [39,40,41,42,43,44,45]. Furthermore, a long-term vitreous replacement by SO could lead to the impairment of normal ions exchanges between the retina and the vitreous, and this could explain the inverse relationship between the SCP VD and the duration of the OS tamponade [43]. 

In addition, a significant CMT increase was observed in patients with intraretinal cysts, considering that half of the eyes filled with SO developed cysts. SO increases the accumulation of pro-inflammatory cytokines in the retro-silicone oil fluid. In fact, in the ocular tissues an inflammatory response happens since macrophages and giant cells with lipid vacuoles have been found [46]. Diffusion of metabolites and cytokines away from the retina was reduced in SO filled eyes [47], leading to the risk of developing intraretinal cysts and CME. No differences were observed in CMT in relation to the type of surgery and the duration of follow-up. 

This study has some limitations. First, a selection bias may have been created because of its retrospective design and one single hospital’s data. There was no randomization for gas or SO tamponade groups. The choice of tamponade was established by the surgeon on the base of multiple aspects, comprising the estimated risk of re-detachment and size of retinal tear. However, the randomization of the choice of tamponade would not be feasible in current practice. We did not differentiate patients for different types of SO used as tamponade. Second, operative time and RD extension can impact on retinal capillary plexuses recovery; however, we did not consider these factors in our study. We did not evaluate the height and volume of the macular detachment, which probably might affect the post-operative macular perfusion outcomes. Third, some segmentation errors and artifacts can persist on the OCT and OCTA images, even though cases with poor quality were excluded. In addition, we did not evaluate OCT structural changes, as well as ellipsoid zone and external limiting-membrane integrity, which are known to be independent factors affecting visual acuity after vitreoretinal surgery.

## 5. Conclusions

In conclusion, the results of this study showed a trend of reduction of macular vascular density in superficial plexus in eyes operated for RRD, with more impressive decreases for Macula-OFF, even following a successful anatomical repair. Despite neither the choice of type of surgery or tamponade differently influencing vascular parameters, in the Macula-ON RRD group the SO tamponade led to the worst final BCVA compared to gas. This suggests a non-ischemic pattern in retinal impairment induced by SO. In addition, the duration of the SO tamponade is correlated with a decrease in VD in the SCP. Therefore, the use of SO should be preferred only in the treatment of complicated retinal detachments with a great importance of an early SO removal, in order to prevent vascular insufficiency in retinal capillary plexuses, also in long-term follow-up. Further investigations are needed with a wider sample and proper randomization of patients to confirm the vascular microarchitecture modifications and to improve the knowledge about vascular relationship with functional recovery.

## Figures and Tables

**Table 1:** Demographic and ocular characteristics of the study participants.

Variables	
Patients, n	82
Age, mean yrs ± SD	63 ± 12
Male, n (%)	50 (61)
Diabetes	6 (7.3)
Hypertension	38 (46.3)
**Pre-operative factors**	
Right eye: Left eye, %	55: 45
Macula OFF, n (%)	49 (59.8)
Pseudophakic	20 (24.4)
**Intraoperative factors**	
Type of surgery, n (%)	
PPV	70 (85.4)
SB	12 (14.6)
Type of surgery	
Gas	65 (79.3)
SO	17 (20.7)
Combined cataract surgery	42 (51.2)
360° laser	12 (14.6)
Operation time, min (mean ± SD)	91.27 ± 34.9
**Post-operative factors**	
Duration of SO tamponade, months (mean ± SD)	4.81 ± 1.8
Hypotony (IOP < 5 mmHg)	0
Secondary glaucoma (IOP > 21 mmHg), n (%)	8 (9.8)
Intraretinal cysts, %	12 (14.6)
Follow-up period (months; m ± SD)	13.1 ± 2

PPV, Pars Plana Vitrectomy; SB, Scleral Buckling; SO, Silicon Oil; IOP, Intraocular Pressure.

**Table 2:** Multivariate linear regression analysis for BCVA. Type of surgery: PPV or SB; Tamponade: gas or SO.

BCVA-Post	Macula-ON Group		Macula-OFF Group	
	β ± SE	*p*-Value	β ± SE	*p*-Value
Type of surgery	−0.009 ± 0.043	0.841	−0.059 ± 0.084	0.487
Tamponade	0.273 ± 0.088	0.004 *	−0.007 ± 0.119	0.955
Follow-up period	0.002 ± 0.005	0.640	−0.001 ± 0.007	0.878

* Statistically significant, *p*-value < 0.05.

**Table 3:** Optical coherence tomography angiography mean values for the Macula-ON and Macula-OFF groups.

	Macula-ON Group			Macula-OFF Group		
	Study Eyes	Control Eyes	*p*-Value	Study Eyes	Control Eyes	*p*-Value
CENTRAL SCP VD	30.20 ± 5.70	32.22 ± 5.66	0.002 *	28.31 ± 5.79	30.32 ± 5.78	0.002 *
INF SCP VD	40.78 ± 4.51	41.80 ± 3.52	0.275	40.33 ± 3.64	42.24 ± 2.38	0.002 *
NAS SCP VD	39.45 ± 4.48	40.90 ± 3.77	0.038 *	38.51 ± 3.73	40.19 ± 3.18	0.010 *
SUP SCP VD	40.32 ± 4.34	42.14 ± 2.78	0.030 *	39.65 ± 3.31	41.42 ± 2.88	0.002 *
TEMP SCP VD	38.42 ± 3.78	39.87 ± 3.35	0.056	38.06 ± 2.85	39.76 ± 2.56	0.000 *
CENTRAL DCP VD	30.30 ± 8.90	30.67 ± 8.15	0.752	28.76 ± 7.44	29.05 ± 8.15	0.784
INF DCP VD	42.76 ± 3.41	42.61 ± 2.65	0.829	42.08 ± 3.08	42.82 ± 2.99	0.185
NAS DCP VD	42.74 ± 3.94	43.00 ± 2.36	0.698	41.54 ± 2.79	42.37 ± 2.93	0.117
SUP DCP VD	42.28 ± 4.06	43.03 ± 2.47	0.307	41.85 ± 2.83	42.40 ± 2.63	0.176
TEMP DCP VD	41.83 ± 3.69	41.96 ± 2.61	0.842	41.43 ± 2.97	41.58 ± 2.70	0.731
CENTRAL SCP VLD	16.17 ± 2.83	17.65 ± 2.86	0.001 *	15.23 ± 2.87	16.53 ±2.96	0.001 *
INF SCP VLD	21.01 ± 3.52	21.70 ± 3.69	0.221	21.11 ± 3.66	22.24 ± 3.35	0.004 *
NAS SCP VLD	20.88 ± 3.33	21.83 ± 3.25	0.086	21.04 ± 3.29	21.92 ± 2.99	0.032 *
SUP SCP VLD	20.55 ± 3.48	21.59 ± 4.04	0.090	20.73 ± 3.27	22.08 ± 3.65	0.001 *
TEMP SCP VLD	20.18 ± 2.98	20.78 ± 3.55	0.259	20.46 ± 2.77	21.50 ± 2.85	0.002 *
CENTRAL DCP VLD	16.54 ± 4.40	16.80 ± 4.58	0.739	16.10 ± 4.25	16.30 ± 4.72	0.766
INF DCP VLD	25.29 ± 3.89	24.66 ± 4.04	0.288	25.06 ± 3.95	25.28 ± 3.90	0.598
NAS DCP VLD	24.66 ± 3.64	25.06 ± 3.72	0.537	24.82 ± 3.60	25.01 ± 3.46	0.655
SUP DCP VLD	24.78 ± 3.74	25.10 ± 4.46	0.600	24.90 ± 3.65	25.41 ± 3.79	0.089
TEMP DCP VLD	24.30 ± 3.17	24.12 ± 3.62	0.747	24.34 ± 3.34	24.26 ± 3.40	0.796

SCP, Superficial Capillary Plexus; DCP, Deep Capillary Plexus,; VD, Vessel Density; VLD, Vessel Length Density; CMT, Central Macular Thickness; in the Macula-ON and Macula-OFF groups. * Statistically significant, *p*-value < 0.05.

## Data Availability

All data will be available on request to the corresponding author.

## References

[1] Cheng K.C., Cheng K.Y., Cheng K.H., Chen K.J., Chen C.H., Wu W.C. (2016). Using optical coherence tomography to evaluate macular changes after surgical management for rhegmatogenous retinal detachment. Kaohsiung J. Med. Sci..

[2] Coppola M., Marchese A., Cicinelli M.V., Rabiolo A., Giuffrè C., Gomarasca S., Querques G., Bandello F. (2020). Macular optical coherence tomography findings after vitreoretinal surgery for rhegmatogenous retinal detachment. Eur. J. Ophthalmol..

[3] Gupta R.R., Iaboni D.S.M., Seamone M.E., Sarraf D. (2019). Inner, outer, and full-thickness retinal folds after rhegmatogenous retinal detachment repair: A review. Surv. Ophthalmol..

[4] Mastropasqua R., Toto L., Mattei P.A., di Nicola M., Zecca I.A.L., Carpineto P., di Antonio L. (2017). Reproducibility and Repeatability of Foveal Avascular Zone Area Measurements Using Swept-Source Optical Coherence Tomography Angiography in Healthy Subjects. Eur. J. Ophthalmol..

[5] de Carlo T.E., Romano A., Waheed N.K., Duker J.S. (2015). A review of optical coherence tomography angiography (OCTA). Int. J. Retin. Vitr..

[6] Spaide R.F., Klancnik J.M., Cooney M.J. (2015). Retinal vascular layers imaged by fluorescein angiography and optical coherence tomography angiography. JAMA Ophthalmol..

[7] Christou E.E., Stavrakas P., Batsos G., Christodoulou E., Stefaniotou M. (2021). Association of OCT-A characteristics with postoperative visual acuity after rhegmatogenous retinal detachment surgery: A review of the literature. Int. Ophthalmol..

[8] Cardillo Piccolino F. (1983). Vascular changes in rhegmatogenous retinal detachment. Ophthalmologica.

[9] Satoh Y. (1989). Retinal circulation in rhegmatogenous retinal detachment demonstrated by videofluorescence angiography and image analysis. I. The condition of retinal circulation before retinal detachment surgery. Nippon Ganka Gakkai Zasshi.

[10] Sato T., Kanai M., Busch C., Wakabayashi T. (2017). Foveal avascular zone area after macula-off rhegmatogenous retinal detachment repair: An optical coherence tomography angiography study. Graefes Arch. Clin. Exp. Ophthalmol..

[11] Wang H., Xu X., Sun X., Ma Y., Sun T. (2019). Macular perfusion changes assessed with optical coherence tomography angiography after vitrectomy for rhegmatogenous retinal detachment. Graefes Arch. Clin. Exp. Ophthalmol..

[12] van Bussel E.M., van der Valk R., Bijlsma W.R., La Heij E.C. (2014). Impact of duration of macula-off retinal detachment on visual outcome: A systematic review and meta-analysis of literature. Retina.

[13] Funatsu R., Terasaki H., Koriyama C., Yamashita T., Shiihara H., Sakamoto T. (2022). Silicone oil versus gas tamponade for primary rhegmatogenous retinal detachment treated successfully with a propensity score analysis: Japan Retinal Detachment Registry. Br. J. Ophthalmol..

[14] Liu Y., Lei B., Jiang R., Huang X., Zhou M., Xu G. (2021). Changes of macular vessel density and thickness in gas and silicone oil tamponades after vitrectomy for macula-on rhegmatogenous retinal detachment. BMC Ophthalmol..

[15] Kang J.W., Yoo R., Jo Y.H., Kim H.C. (2017). Correlation of microvascular structures on optical coherence tomography angiography with visual acuity in retinal vein occlusion. Retina.

[16] Williams G.A., Reeser F., O’Brien W.J., Fleischman J.A. (1983). Prostacyclin and thromboxane A2 derivatives in rhegmatogenous subretinal fluid. Arch. Ophthalmol..

[17] Quintyn J.C., Brasseur G. (2004). Subretinal fluid in primary rhegmatogenous retinal detachment: Physiopathology and composition. Surv. Ophthalmol..

[18] Ricker L.J., Kijlstra A., de Jager W., Liem A.T., Hendrikse F., La Heij E.C. (2010). Chemokine levels in subretinal fluid obtained during scleral buckling surgery after rhegmatogenous retinal detachment. Investig. Ophthalmol. Vis. Sci..

[19] Roldán-Pallarés M., Musa A.S., Hernández-Montero J., Bravo-Llatas C. (2009). Preoperative duration of retinal detachment and preoperative central retinal artery hemodynamics: Repercussion on visual acuity. Graefes Arch. Clin. Exp. Ophthalmol..

[20] Iandiev I., Uhlmann S., Pietsch U.C., Biedermann B., Reichenbach A., Wiedemann P., Bringmann A. (2005). Endothelin receptors in the detached retina of the pig. Neurosci. Lett..

[21] Çetinkaya Yaprak A., Küçük M.F., Yaprak L., Erol M.K. (2021). Change in retinal and choroidal microvascular structures after rhegmatogenous retinal detachment surgery and effects on visual recovery. J. Fr. Ophtalmol..

[22] McKay K.M., Vingopoulos F., Wang J.C., Papakostas T.D., Silverman R.F., Marmalidou A., Lains I., Eliott D., Vavvas D.G., Kim L.A. (2020). Retinal Microvasculature Changes After Repair of Macula-off Retinal Detachment Assessed with Optical Coherence Tomography Angiography. Clin. Ophthalmol..

[23] Chatziralli I., Theodossiadis G., Parikakis E., Chatzirallis A., Dimitriou E., Theodossiadis P. (2020). Inner retinal layers’ alterations and microvasculature changes after vitrectomy for rhegmatogenous retinal detachment. Int. Ophthalmol..

[24] Tsen C., Sheu S., Chen S., Wu T. (2019). Imaging analysis with optical coherence tomography angiography after primary repair of macula-off rhegmatogenous retinal detachment. Graefes Arch. Clin. Exp. Ophthalmol..

[25] D’Aloisio R., Gironi M., Verdina T., Vivarelli C., Leonelli R., Mariotti C., Kaleci S., Toto L., Mastropasqua R. (2022). Early Structural and Vascular Changes after Within-24 Hours Vitrectomy for Recent Onset Rhegmatogenous Retinal Detachment Treatment: A Pilot Study Comparing Bisected Macula and Not Bisected Macula. J. Clin. Med..

[26] Hong E.H., Cho H., Kim D.R., Kang M.H., Shin Y.U., Seong M. (2020). Changes in Retinal Vessel and Retinal Layer Thickness After Vitrectomy in Retinal Detachment via Swept-Source OCT Angiography. Investig. Ophthalmol. Vis. Sci..

[27] Barca F., Bacherini D., Dragotto F., Tartaro R., Lenzetti C., Finocchio L., Virgili G., Caporossi T., Giansanti F., Savastano A. (2020). OCT Angiography Findings in Macula-ON and Macula-OFF Rhegmatogenous Retinal Detachment: A Prospective Study. J. Clin. Med..

[28] Jiang J., Chen S., Jia Y.D., Li R., Zhou J.X., Li R.M. (2021). Evaluation of macular vessel density changes after vitrectomy with silicone oil tamponade in patients with rhegmatogenous retinal detachment. Int. J. Ophthalmol..

[29] Bonfiglio V., Ortisi E., Scollo D., Reibaldi M., Russo A., Pizzo A., Faro G., Macchi I., Fallico M., Toro M.D. (2019). Vascular changes after vitrectomy for rhegmatogenous retinal detachment: Optical coherence tomography angiography study. Acta Ophthalmol..

[30] Hassanpoor N., Milani A.E., Kordestani A., Niyousha M.R. (2021). Analysis of Retinal Layers’ Thickness and Vascular Density after Successful Scleral Buckle Surgery. J. Curr. Ophthalmol..

[31] Nam S.H., Kim K., Kim E.S., Kim D.G., Yu S.Y. (2021). Longitudinal Microvascular Changes on Optical Coherence Tomographic Angiography after Macula-Off Rhegmatogenous Retinal Detachment Repair Surgery. Ophthalmologica.

[32] Resch M.D., Balogh A., Lászik G., Nagy Z.Z., Papp A. (2021). Association between retinal vessel density and postoperative time after primary repair of rhegmatogenous retinal detachment. PLoS ONE.

[33] Borrelli E., Balasubramanian S., Triolo G., Barboni P., Sadda S.R., Sadun A.A. (2018). Topographic Macular Microvascular Changes and Correlation With Visual Loss in Chronic Leber Hereditary Optic Neuropathy. Am. J. Ophthalmol..

[34] Borrelli E., Toto L., Viggiano P., Evangelista F., Palmieri M., Mastropasqua R. (2020). Widefield topographical analysis of the retinal perfusion and neuroretinal thickness in healthy eyes: A pilot study. Eye.

[35] Agarwal A., Aggarwal K., Akella M., Agrawal R., Khandelwal N., Bansal R., Singh R., Gupta V., OCTA Study Group (2019). Fractal dimension and optical coherence tomography angiography features of the central macula after repair of rhegmatogenous retinal detachments. Retina.

[36] Kim K., Kim E.S., Yu S.Y. (2018). Optical coherence tomography angiography analysis of foveal microvascular changes and inner retinal layer thinning in patients with diabetes. Br. J. Ophthalmol..

[37] Menke M.N., Kowal J.H., Dufour P., Wolf-Schnurrbusch U.E., Ceklic L., Framme C., Wolf S. (2014). Retinal layer measurements after successful macula-off retinal detachment repair using optical coherence tomography. Investig. Ophthalmol. Vis. Sci..

[38] Lee J.Y., Kim J.Y., Lee S.Y., Jeong J.H., Lee E.K. (2020). Foveal Microvascular Structures in Eyes with Silicone Oil Tamponade for Rhegmatogenous Retinal Detachment: A Swept-source Optical Coherence Tomography Angiography Study. Sci. Rep..

[39] Williams P.D., Fuller C.G., Scott I.U., Fuller D.G., Flynn H.W. (2008). Vision loss associated with the use and removal of intraocular silicone oil. Clin. Ophthalmol..

[40] Dogramaci M., Williams K., Lee E., Williamson T.H. (2013). Foveal light exposure is increased at the time of removal of silicone oil with the potential for phototoxicity. Graefes Arch. Clin. Exp. Ophthalmol..

[41] García-Ayuso D., Salinas-Navarro M., Agudo-Barriuso M., Alarcón-Martínez L., Vidal-Sanz M., Villegas-Pérez M.P. (2011). Retinal ganglion cell axonal compression by retinal vessels in light-induced retinal degeneration. Mol. Vis..

[42] Grzybowski A., Pieczynski J., Ascaso F.J. (2014). Neuronal complications of intravitreal silicone oil: An updated review. Acta Ophthalmol..

[43] Winter M., Eberhardt W., Scholz C., Reichenbach A. (2000). Failure of potassium siphoning by Müller cells: A new hypothesis of perfluorocarbon liquid-induced retinopathy. Investig. Ophthalmol. Vis. Sci..

[44] Inoue M., Iriyama A., Kadonosono K., Tamaki Y., Yanagi Y. (2009). Effects of perfluorocarbon liquids and silicone oil on human retinal pigment epithelial cells and retinal ganglion cells. Retina.

[45] Bambas B., Eckardt C., Vowinkel E., Kruse H. (1995). Toxische Substanzen im Silikonöl nach intraokularer Injektion [Toxic substances with silicone oil after intraocular injections]. Ophthalmologe.

[46] Wickham L., Asaria R.H., Alexander R., Luthert P., Charteris D.G. (2007). Immunopathology of intraocular silicone oil: Enucleated eyes. Br. J. Ophthalmol..

[47] Asaria R.H., Kon C.H., Bunce C., Sethi C.S., Limb G.A., Khaw P.T., Aylward G.W., Charteris D.G. (2004). Silicone oil concentrates fibrogenic growth factors in the retro-oil fluid. Br. J. Ophthalmol..

